# What Do True Gender Ratios and Stereotype Norms Really Tell Us?

**DOI:** 10.3389/fpsyg.2016.01036

**Published:** 2016-07-07

**Authors:** Pascal M. Gygax, Alan Garnham, Sam Doehren

**Affiliations:** ^1^Department of Psychology, University of FribourgFribourg, Switzerland; ^2^School of Psychology, University of SussexBrighton, UK; ^3^Horizon Center for Doctoral Training, University of NottinghamNottingham, UK; ^4^School of Psychology, University of NottinghamNottingham, UK

**Keywords:** interpretation, stereotypes, role names, ratings, true ratios, archival sources

## Abstract

We present a Focused Review on work that was conducted to compare perceived distributions of men and women in occupations and other social roles with actual real world distributions. In previous work, we showed that means for the two sources were similar and the **correlation** between them was high. However, in the present paper, although we argue that comparing subjective **gender stereotype norms** and real world data about gender ratios is an interesting endeavor, we also discuss the limits to and difficulties in trying to determine the **causal relationship** between them. Most crucially, we argue that our data does not allow us to deduce with certainty that subjective gender norms are based directly on gender ratios.

## Introduction

Psychologists, from a number of subdisciplines, have been interested in a wide variety of questions about stereotyping. Psycholinguists have addressed a particular set of questions (e.g., Gygax et al., 2008), different in focus from those of social psychologists, about the use of stereotype information (or rather a particular aspect of stereotype information) to inform the representation of characters introduced into discourse or text, using terms that are stereotyped for gender. The particular aspect of stereotyping that psycholinguists are interested in has been people's knowledge, or rather their beliefs, about whether certain occupations or roles are typically filled by women or by men. There are various established ways of investigating people's beliefs about such matters, mostly by asking them direct questions about those beliefs. But there remains the question of how accurate those beliefs are. If they are relatively accurate, then holding them need not reflect some of the more negative aspects of stereotyping that other psychologists study. Indeed, accurate information about the actual state of the world should be a better basis than inaccurate information for instigating change, if it is thought desirable.

In order test the veracity of such “stereotype” beliefs, Garnham et al. (2015) gathered data on actual female:male ratios for more than 200 occupations and social roles. These occupations and social roles were taken from a set of 422 terms for which we had collected **stereotype norms** in an earlier study (Misersky et al., 2014).

The **gender stereotypes** we are interested in, sometimes referred to as conceptual gender, derive from *generalized beliefs* about occupational or social roles that are more or less likely to be held by one gender or the other. One set of issues about such beliefs relates to their nature and construction. For our purposes we focus on one particular aspect of those beliefs—the actual ratio of females to males fulfilling those roles. There are important issues about, broadly speaking, attitudes to those beliefs. Do people think that the ratios they believe to hold are the ones that should hold? We are not directly concerned with such questions, but one important issue in assessing people's attitudes to those beliefs is whether those beliefs are correct. It is this particular issue that we addressed in Garnham et al. (2015).

KEY CONCEPT 1 **Gender stereotypes (of occupations).**

Opinions or estimates that derive from generalized beliefs about occupational or social roles, and whether they are more or less likely to be undertaken by one gender or the other. Gender stereotypes may contain prejudicial components.

Our original interest, as psycholinguists, outlined in earlier publications (see especially Carreiras et al., 1996; Garnham et al., 2002, 2012; Oakhill et al., 2005; Gygax et al., 2008, 2009), was to understand the relatively spontaneous use of stereotype information in a number of cognitive as well as social mental processes. In particular, we examined the influence of **gender stereotypes** when readers, or more broadly language comprehenders, build a mental representation of a person in a specific occupation, or social role. For example, in a study on the spontaneous representation of gender, Gygax et al. (2008) investigated what gender people would mentally represent when reading sentences such as:

(a) The musicians were walking through the station.

In their study, they contrasted sentences such as (a), where it is unlikely that a shared generalized belief would direct comprehenders into representing a specific gender, with sentences such as (b), where previously collected **stereotype norms** indicate that comprehenders should be more likely to mentally represent, or imagine, women.

(b) The nurses were walking through the station.

In English, at least (experiments were also conducted in French and German), the authors showed that readers' representation of gender was heavily based on the role noun's stereotypicality. Concretely, readers showed some difficulty in processing continuation sentences such as (c) when it followed (b), but not (a).

(c) Since sunny weather was forecast several of the *men* weren't wearing a coat.

More generally, research on this topic (using a wide range of methods, from judgment tasks to EEG) have shown that in English, where there is no grammatical gender information on most nouns, (1) readers base their mental representations of gender on stereotypes (e.g., Oakhill et al., 2005), (2) the mechanisms leading them to do so are difficult to overcome, though (3) certain specific types of training may lead readers to distant themselves from habitual stereotypes (Finnegan et al., 2015a,b). In other languages, stereotype information interacts with grammatical gender to produce more complex, but readily explicable, patterns of behavior.

From a psycholinguistic point of view, the results of these studies are straightforward. Generalized beliefs about female and male occupancy of various roles, as measured in norming studies, determine, in conjunction with grammatical gender information, in languages in which it is present, whether characters mentioned using role names are taken to be female or male. From a broader perspective, however, various questions remain, and in particular the nature of the stereotypical belief measured in norming studies. Given that inferences about gender, when encountering an occupational or social role, are primarily driven by people's beliefs about gender representation in that roles, the question arises as to what exactly constitutes those beliefs. On the one hand, these beliefs might be derived directly from observation of probabilities, in which case they might provide a reasonably accurate representation of reality. On the other hand, they might be influenced by shared social norms, learned, for example, through social media, television or school, by prejudice, or by other factors not closely linked to actual occupancy of the roles. In this case beliefs about gender may be associated with the kind of *biases* associated with the negative aspects of stereotyping, and that inevitably direct comprehenders' representations toward a particular gender.

Before pursuing this complex question, it is important to understand how **stereotype norms** are usually measured in studies such as Misersky et al. (2014), and in what way these norming studies are typically carried out as a prelude to psycholinguistic experiments in which stereotypical gender is manipulated.

## Acquiring gender stereotype norms for psycholinguistic research

The psycholinguistic research on stereotyping in which we have been engaged, going back to Carreiras et al. (1996), was motivated not by questions about stereotyping *per-se*, but by questions about inference in text comprehension. The importance of inference in text comprehension was first emphasized by Bransford's (e.g., Bransford et al., 1972) characterization of comprehension as an integrative and a constructive process. By integrative, Bransford meant that information in different parts of a text needed to be put together in often quite complex ways. By constructive he meant that information explicit in a text had to be combined with other information, often background knowledge, to get a full interpretation of the text. Integration and construction often go hand in hand, and both can require inferences to be made.

Bransford's own writings suggested that many inferences were made as texts are read, but much of his evidence came from what happened after reading was complete (e.g., in answering questions about the text), and was later dismissed as not providing direct evidence for what happens during reading. Many people pointed out that there was no limit to the number of inferences that might be made from any particular text, and various ideas were propounded for how those inference might be limited. One suggestion of a set of inferences that might be made was for those based, or based primarily, on the presence of a single word in the text. Initially, it was proposed that the key set of inferences were those based on the core meaning of a word. For example, *dress* means to put clothes on, and Garrod and Sanford (1981) were able to show that clothes were as much a part of the representation of “Mary dressed the baby” as of “Mary put the clothes on the baby.” Our early work on stereotypes showed a similar finding for words such as “nurse” and “engineer,” where an inference to the probable gender of a person described as “the nurse” or “the engineer” seemed to be made immediately. In relation to theories of inference, the interesting aspect of this result is that, for example, being female is in fact not part of the core definition of *nurse*.

To carry out these studies we needed normative information about stereotyping of occupations and social roles in the population we were studying. Because we were not directly interested in the prejudicial aspects of stereotyping, we asked people directly about their beliefs about the relative numbers of females and males in the kinds of roles we wanted to study. *Prima facie*, therefore, we might have expected to tap relatively accurate beliefs about these ratios, provided that people had some reasonably accurate way of estimating them. We have used similar methods in later studies, including our most recent, large scale study reported in Misersky et al. (2014). For these norms, we assembled an extensive list of English role nouns (*N* = 422) and translated as many as possible into six other languages (Czech, French, German, Italian, Norwegian, and Slovak), which were represented in the research network of which we were part (Marie Curie ITN, Language, Cognition, and Gender, ref no…). These norms were intended for use in the selection of stimulus materials for studies on gender representations. To collect the norms, we used an on-line questionnaire, in which participants were presented with the role nouns, each accompanied by an 11-point scale (*0% women/100% men* to *100%women/0%men*^1^). Participants, in all languages were instructed as follows:

“*Your task is to estimate to what extent the groups are made up of women or men. For instance, if you associate the group “actors” exclusively with women, click the button that corresponds to “100% women” (“actresses”). If you think that the group is formed of men only, click the button that corresponds to “100% men” (“actors”). If you think that the group is formed of an equal percentage of men and women, click the button on the middle (50/50). Use the other circles to represent other percentages as appropriate. There are no right or wrong answers. We are interested in what you think is the real proportion of men and women in the social groups and not in your view of gender equality. Please answer as quickly as possible, without thinking too much about the meaning of each group.”*

These instructions demonstrate that we specifically asked people to make a judgment about how they believe things are in the world, not how they should be. Furthermore, they do not ask about people's personal views about gender equality, only about their beliefs about the proportion of men and women in the different occupations.

## Examining true gender ratio

Of course, it does not follow that if you ask people to report their beliefs about how the world is, either that they will comply with the instruction, or, even if they do, that their reported beliefs will be accurate. In some ways, these issues are unimportant in a narrow interpretation of psycholinguistic results. People's beliefs drive their interpretation of texts, and it does not matter, in this narrow sense, whether those beliefs are correct. However, the question of whether people's beliefs about the occupancy of social roles are correct does have implications for whether their “stereotypes” might, for instance, be benign or pernicious. Beliefs that accurately reflect the state of the world are not necessarily benign. Nevertheless, the accuracy, or otherwise, of people's beliefs may have implications for how we interpret those beliefs.

In Garnham et al. (2015), we wanted to compare the norms, or beliefs, reported in Misersky et al. (2014) to actual true gender ratios. In the first instance we focused on the norms for English and **true gender ratios** in the UK. There are many reasons why such a comparison might be of interest. One reason, which we mentioned in the paper, would be to determine whether there was evidence that stereotyping is based on outdated **true gender ratios** (Wilbourn and Kee, 2010), with, for example, more males being reported as occupying traditionally male occupations than is currently the case, or on (possibly incorrect) assumptions about current female/male ratios (Lopez-Zafra and Garcia-Retamero, 2012; Mills et al., 2012), which might, for example, make the estimates generally inaccurate. Because of the way we approached the problem, our research was constrained by the list of 422 English role names in the Misersky et al. list. Largely for this reason, it proved difficult to obtain some of the **true gender ratios** we were seeking. The categories used in our principal source, data from the UK Office for National Statistics, did not always map easily onto the terms used in the Misersky et al. study. Other issues with this data source and with other data sources, such as Scopus and Google Scholar, are detailed in Garnham et al. (2015). In the end, we obtained gender ratios for 290 of the 422 terms, but 85 of these were clearly problematic, for reasons that are, again, detailed in the original paper. Nevertheless, we had estimates of gender ratios, based on a satisfactory mapping between Misersky et al.'s terms and those for which we had real world data, for a little less than half of the occupations normed in Misersky et al., which amounted to 205 terms in total. For these 205 role names, the **correlation** between the true ratio and the ratio from the norms was 0.849. For the 85 excluded items, the **correlation** was much lower at 0.273.

KEY CONCEPT 2 **True gender ratios.**

The ratio of men to women (or vice versa) in social roles or occupations. Such ratios should be derived from reliable statistical data, and will depend on context (e.g., surgeons in the UK).

KEY CONCEPT 3 **Correlation.**

A symmetrical relationship between two or more variables that change together. A correlation may be positive, when the two variables increase or decrease together, or negative, when they change in different directions. Correlation is not evidence for a causal relationship, though causes and effects do co-vary.

The high positive **correlation** simply means that the two sets of scores increase together quite closely. However, it does not mean that the judgments are accurate. In regression terms, either the slope or the intercept could be inaccurate. For the 205 roles names for which we had reliable data, the Misersky et al. ratios were on average 0.02 greater than the true ratios, indicating a close match. However the slope of the regression of the Misersky ratios on the true ratios was 0.48, indicating—taking into account the accurate mean—a tendency for judgments to be less extreme than the true values. More specifically, the regression equation was:

$$\begin{array}{l} \text{Misersky ratio }=0.242\;+\;0.479\;\times \text{True Ratio} \end{array}$$

So, at the mid-point, where the true ratio is 0.5, the predicted Misersky ratio is 0.481, and both high and low ratios are predicted to be less extreme than the actual values.

In Garnham et al. (2015) we pointed out a problem with reported true ratios of 1 or 0. Such ratios were (apparently) reported in ONS statistics when the number of women or men in an occupation was numerically small. Depending on how many women or men were in larger category, the effect of treating the smaller category as being 0 could have a larger or a smaller effect. In the most extreme case, the actual ratio of female to male shoemakers could have been anywhere between 0 and 0.43 (see original paper for details). If we remove these items, the slope for the remaining 154 items increases somewhat and the equation becomes:

$$\begin{array}{l} \text{Misersky ratio }=0.214\;+\;0.564\;\times \text{ True Ratio} \end{array}$$

The predicted judgment for a true ratio of 0.5 is 0.496, which is even close to the real ratio, and there is slightly less attenuation in the prediction of the more extreme scores.

These observations raise two questions. The first is how the **correlation** between judged ratios and true ratios comes about. The second is how the details of relationship observed relate to a broader notion of stereotyping. We believe that the answers to these questions are entwined in a complicated way.

## Is there a causal relation between stereotype norms and true gender ratios?

It is a truism that a **correlation** is not necessarily an indication of a **causal relationship**. For one thing, **correlation** is a symmetrical relationship, whereas causation is not. For another, two effects can be correlated because they are both caused by the same factors, in a simple or a complex way. Nevertheless, it is notable that, in our data, there appears to be no systematic bias in estimating gender ratios. The average difference between true ratios and estimated ratios is close to zero. However, the fact that the regression slope is closer to a half than to one suggests that the absolute deviations between the two scores increases as the proportions of females and males become more extreme, whether in favor of females or in favor of males. If anything, the judged ratios are not as extreme (or “stereotyped”) as the real ratios. So, although estimated gender ratios could be relatively straightforwardly derived from knowledge of true ratios, however obtained, there must at least be some other influence to account for the attenuation in the extreme values in the estimates. This influence could in part derive from the method of obtaining judgments. For example, it is plausible that people would be reluctant to say a role is 100% female or 100% male. But deciding against this response requires, given the way our questionnaire is constructed, selection of a 10% male or 10% female response, which would not be an accurate reflection of a role in which, for example, 98% of the occupants were male. However, the predicted judged value for a role with 100% men is 89% men, so the method of measurement cannot explain all of the attenuation at extreme values.

KEY CONCEPT 4 **Causal relationship.**

An asymmetrical relationship in which one type of event, state or process (the cause) brings about the other (the effect).

One consequence of the nature of the relation between **true gender ratios** and judged gender ratios, and in particular the apparent lack of bias in the set of judgments as a whole, is that one might be tempted to say that the judgments collected by Misersky et al. do not truly constitute stereotypes, or at least that they not reflect stereotyping by the person making the judgments. To make this point is not to say that people are not disadvantaged by being (partially) excluded from certain roles, only that people's judgments of the number of people in those roles do not reflect any additional bias, other than the biases that determine whether women or men fill the roles.

It is worth emphasizing that the fact that **stereotype norms** deviate from real world ratios only rules out a very direct link between the two. The more important conclusion from our findings is that there appears to be a common foundation for real world gender ratios and our beliefs about them. Indeed, given the fact that most people do not have access to reliable statistical information, or at least that they do not typically spend time poring through data from the Office of National Statistics, or similar sites in other countries, people seem remarkably well-tuned to the relative female:male ratios in a wide ranges of occupations and social roles.

However, it is also true that our data cannot rule out the possibility that people's judgments are determined by prejudicial processes, which might in turn shape reality. An extreme version of this idea would be that people's judgments reflect directly how they think things should be, and that the prejudices reflected in such judgments also determine whether people take on certain occupations and roles. This extreme version is somewhat unlikely, given that it would require people in the norming studies to explicitly go against the instructions they are given to make judgments based on how things are rather than how they ought to be. Nevertheless, if judgments in the Misersky et al. task and real gender ratios are both the result of a complex set of processes, broadly prejudicial effects could be at least partially responsible for the pattern of results we obtained. For example, through shared beliefs, reflected in, or even manipulated by, the media, through education, or through parenting, people may be more inclined to choose particular occupations. At the same time, they may also believe that these occupations are particularly fitted for a specific gender, and their judgments of who takes up such occupations may be influenced by these factors. Such processes may be pernicious, particular if they have their effects early in people's lives. For example, in many cultures, women are attributed less mathematical skills than men (e.g., Spelke, 2005). Interestingly, it has been reported that gender differences in performance in mathematics are non-existent in childhood (or very small; Hyde, 2005). Thus, there is a common discriminatory belief that may have two consequences. First, the belief becomes lodged in people's minds, and may affect, either positively or negatively, their feelings about their own mathematical ability. Second, girls and women may be reluctant to choose careers associated with mathematics. Both the judgments about *mathematicians* in Misersky et al. (2014), and the real world ratio reported in Garnham et al. (2015), may reflect these societal attitudes to women and mathematics.

This kind of *indirect link* explanation for our findings does not fit well with the idea that the norms collected should not be thought of as stereotypes because they reflect *reality*. It remains true that the judgments do not reflect any additional bias above what is seen in the distribution of females and males in various roles. Nevertheless, on this view, the beliefs that underlie the judgments are part of what produces this prejudicial distribution, and in this sense they stereotype people, in the negative sense of stereotyping. However, on neither view does the fact that our real world data tells us that there are more male mathematicians than female mathematicians mean that mathematics is a domain particularly fitted, biologically or otherwise, for boys.

As should be clear from our discussion above, neither the data from the norming studies nor the real world ratios reported by Garnham et al. allow us to distinguish among the possible causal links between norming and real world data. However, the studies raise some interesting questions about our mental representations of gender, and provide some constraints on what the answers to those questions must be.

## Norms and/or real world data as biases

One issue that we have not considered so far is that, although our data show that people are sensitive to the ratio of females to males who filled particular roles, when they read about a surgeon or some surgeons, they appear to use “stereotype” information to infer that the surgeon or surgeons are male, regardless of whether the proportion of male surgeons (or whatever) is rather high or very high.

As we mentioned in the Introduction, adult readers have some difficulty with sentences such as “The surgeons were walking through the station. Since sunny weather was forecast several of the women weren't wearing a coat.” (Gygax et al., 2008). This difficulty is interpreted as showing that when encountering the role noun *surgeon*, readers form a male mental representation. Reading of later parts of the text can be impaired by this specific representation, if, as in the example, some or all of the surgeons turn out to be female. In some cases more extreme effects are seen, and readers cannot form a coherent representation of the information in a text, simply because they do not consider the possibility that a surgeon is a woman. For example, Reynolds et al. (2006) found that many people could not resolve the following well-known riddle:

*This morning a father and his son were driving along the motorway to work, when they were involved in a horrible accident. The father was killed and the son was quickly driven to hospital severely injured. When the boy was taken into the hospital a passing surgeon exclaimed: “Oh my God, that is my son!*”

If people used probabilistic information based on the fact that most, but not all, surgeons are male, they should have been able to see that the surgeon could be a woman. In this case, they may have been slightly delayed in solving the riddle, as Gygax et al.'s participants were slowed down in figuring out that there were women in the group of surgeons. However, they often tried to reject the more explicitly stated, and less contravertable, information that the surgeon was the parent of the boy, rather than the probabilistic information that a surgeon is more likely to be male than female. Even if people have reasonably accurate information about females and males who occupy certain roles, they can display biases in using that information that lead them to incorrect conclusions.

A more complex illustration of bias is seen in a study by Vervecken et al. (2015) on an adolescent population. In their study, Vervecken et al. presented 15 occupations^2^ to adolescents aged 12–17, and asked them to indicate on a five-point Likert scale who they thought would *succeed* in each occupation (1 = *only men*, 3 = *men and women alike*, 5 = *only women*). Thus, the authors did not simply ask what they thought the proportions of men and women were, but who they thought *would succeed* in these jobs. Their results were in line with Misersky et al.'s data, demonstrating that beliefs about the proportion of men and women in different occupations is associated with beliefs about the success of women and men in those occupations. In other words, whether their representations of gender were based on shared beliefs or on true gender ratios, these adolescents associated the ratio of female to male in professions with success in those professions. As far as we know, there is no evidence for such a link, but whether there is, is an empirical question, given a precise enough definition of success. Of course, an exhaustive approach to this question should address these effects as potentially age-dependent.

## Conclusions

Comparing subjective **gender stereotype norms** and real world data about gender ratios in occupations and social roles is an interesting endeavor, and raises some stimulating issues, especially for those interested in the way beliefs about certain occupations constrain our representations of gender. However, it is also important to understand that the **causal relationship** between the two may take a variety of forms, and our analysis can only impose a limited number of constraints on what that relationship might be. Importantly, it cannot be said with certainty that subjective gender norms are straightforwardly based on true gender ratios, inasmuch as both may derive from a system of beliefs and their effect on behavior, rather than one directly causing the other.

KEY CONCEPT 5 **(Gender) stereotype norms.**

Information gathered by directly asking people for their beliefs about the relative numbers of females and males in certain roles or occupations. In our research we specifically ask people to base their responses on beliefs about how the world is, not how they think it should be, but norms can be collected in other ways.

## Author contributions

All three authors participated in the initial study, for which we were asked to write a Focused Review. PG wrote the first draft of the paper, and both AG and SD participated in commenting and re-writing parts of the Focused Review.

### Conflict of interest statement

The authors declare that the research was conducted in the absence of any commercial or financial relationships that could be construed as a potential conflict of interest.

## Footnotes

^1^The order of presentation of the stimuli was random for each participant, and the order of the scale (i.e., women on the left, women on the right) was counterbalanced.

^2^The experimental manipulation of Vervecken et al. goes beyond the scope of this paper, as it concerned mainly French.

## References

[B1] Bransford J. D. Barclay J. R. Franks J. J. (1972). Sentence memory: a constructive versus interpretive approach. Cogn. Psychol. 3, 193–209. 10.1016/0010-0285(72)90003-5

[B2] Carreiras M. Garnham A. Oakhill J. Cain K. (1996). The Use of stereotypical gender information in constructing a mental model: evidence from English and Spanish. Q. J. Exp. Psychol. A. 49, 639–663. 10.1080/713755647 8828401

[B3] Finnegan E. Garnham A. Oakhill J. (2015a). Social-consensus feedback as a strategy to overcome spontaneous gender stereotypes. Discourse Process 52, 434–462. 10.1080/0163853X.2015.1026680

[B4] Finnegan E. Oakhill J. Garnham A. (2015b). Counter-stereotypical pictures as a strategy for overcoming spontaneous gender stereotypes. Front. Psychol. 6:1291. 10.3389/fpsyg.2015.01291 26379606 PMC4550774

[B5] Garnham A. Doehren S. Gygax P. (2015). True gender ratios and stereotype rating norms. Front. Psychol. 6:1023. 10.3389/fpsyg.2015.01023 26257681 PMC4510832

[B6] Garnham A. Gabriel U. Sarrasin O. Gygax P. Oakhill J. (2012). Gender representation in different languages and grammatical marking on pronouns: when beauticians, musicians, and mechanics remain men. Discourse Process 49, 481–500. 10.1080/0163853X.2012.688184

[B7] Garnham A. Oakhill J. V. Reynolds D. J. (2002). Are inferences from stereotyped role names to characters' gender made elaboratively? Mem. Cogn. 30, 439–446. 10.3758/BF03194944 12061764

[B8] Garrod S. C. Sanford A. J. (1981). Bridging inferences and the extended domain of reference, in Attention and Performance IX, eds Long J. Baddeley A. (Hillsdale, NJ: Lawrence Erlbaum Associates Inc), 331–346

[B9] Gygax P. Gabriel U. Sarrasin O. Oakhill J. V. Garnham A. (2008). Generically intended, but specifically interpreted: when beauticians, musicians and mechanics are all men. Lang. Cogn. Process 23, 464–485. 10.1080/01690960701702035

[B10] Gygax P. Gabriel U. Sarrasin O. Oakhill J. V. Garnham A. (2009). Some grammatical rules are more difficult than others: the case of generic interpretation of masculine. Eur. J. Psychol. Educ. 24, 235–246. 10.1007/BF03173014

[B11] Hyde J. S. (2005). The gender similarities hypothesis. Am. Psychol. 60, 581–592. 10.1037/0003-066X.60.6.581 16173891

[B12] Lopez-Zafra E. Garcia-Retamero R. (2012). Do gender stereotypes change? Dyn. Gend. Stereotypes Spain J. Gend. Stud. 21, 169–183. 10.1080/09589236.2012.661580

[B13] Mills M. J. Culbertson S. S. Huffman A. H. Connell A. R. (2012). Assessing gender biases: development and initial validation of the gender role stereotypes scale. Gend. Manag. 27, 520–540. 10.1108/17542411211279715

[B14] Misersky J. Gygax P. Canal P. Gabriel U. Garnham A. Braun F.. (2014). Norms on the gender perception of role nouns in Czech, English, French, German, Italian, Norwegian, and Slovak. Behav. Res. Methods 46, 841–871. 10.3758/s13428-013-0409-z 24163213

[B15] Oakhill J. V. Garnham A. Reynolds D. J. (2005). Immediate activation of stereotypical gender information in reading. Mem. Cogn. 33, 972–983. 10.3758/BF03193206 16496719

[B16] Reynolds D. J. Garnham A. Oakhill J. (2006). Evidence of immediate activation of gender information from a social role name. Q. J. Exp. Psychol. 59, 886–903. 10.1080/02724980543000088 16608753

[B17] Spelke E. S. (2005). Sex differences in intrinsic aptitude for mathematics and science. Am. Psychol. 60, 950–958. 10.1037/0003-066X.60.9.950 16366817

[B18] Vervecken D. Gygax P. Gabriel U. Guillod M. Hannover B. (2015). Warm businessmen, cold housewives? Effects of gender-fair language on adolescents' perceptions of occupations. Front. Psychol. 6:1437 10.3389/fpsyg.2015.01437 PMC4585286 26441805

[B19] Wilbourn M. P. Kee D. W. (2010). Henry the nurse is a doctor Too: implicitly examining children's gender stereotypes for male and female occupational roles. Sex Roles 62, 670–683. 10.1007/s11199-010-9773-7

